# Perspectives of complementary and alternative medicine (CAM) practitioners in the support and treatment of infertility

**DOI:** 10.1186/1472-6882-14-394

**Published:** 2014-10-14

**Authors:** Erin O’Reilly, Marika Sevigny, Kelley-Anne Sabarre, Karen P Phillips

**Affiliations:** Interdisciplinary School of Health Sciences, Faculty of Health Sciences, University of Ottawa, 25 University Private, Room 138, Ottawa, Ontario K1N 6N5 Canada

**Keywords:** Infertility, Qualitative, Complementary and alternative medicine, Naturopathy, Traditional Chinese medicine, Acupuncture

## Abstract

**Background:**

Infertility patients are increasingly using complementary and alternative medicine (CAM) to supplement or replace conventional fertility treatments. The objective of this study was to determine the roles of CAM practitioners in the support and treatment of infertility.

**Methods:**

Ten semi-structured interviews were conducted in Ottawa, Canada in 2011 with CAM practitioners who specialized in naturopathy, acupuncture, traditional Chinese medicine, hypnotherapy and integrated medicine.

**Results:**

CAM practitioners played an active role in both treatment and support of infertility, using a holistic, interdisciplinary and individualized approach. CAM practitioners recognized biological but also environmental and psychosomatic determinants of infertility. Participants were receptive to working with physicians, however little collaboration was described.

**Conclusions:**

Integrated infertility patient care through both collaboration with CAM practitioners and incorporation of CAM’s holistic, individualized and interdisciplinary approaches would greatly benefit infertility patients.

## Background

Infertility is a complex, multifactorial condition characterized by the absence of conception following one year of unprotected sexual intercourse [1–3]. Biological, genetic [4], infectious [5], lifestyle [6–9] and environmental [10, 11] risk factors are associated with both male and female infertility. Fertility issues are primarily investigated by family practice physicians and gynecologists (i.e. diagnostic investigations, endocrine disorders, anovulatory conditions) with unresolved infertility ultimately treated by reproductive endocrinologists using assisted reproductive technologies (ART) [2, 3]. For some patients, ART presents significant financial, psychological, moral and ethical challenges which may lead to discontinuation of treatment [12]. As medicine, in particular ART, becomes increasingly technological, patients are choosing complementary and alternative medicine (CAM); perceived as more natural with less side effects [13, 14]. CAM provides non-mainstream approaches which complement or replace conventional medicine [15]. Acupuncture, hypnotherapy, chiropractic and osteopathic manipulation, naturopathy, homeopathy and traditional Chinese medicine (TCM) are examples of CAM [15].

In Canada, CAM use for all conditions is increasing, with the typical patient female aged 20-64 years [16, 17]. Treatment of infertility using CAM has been reported in studies from Australia [18–23], Canada [24], United Kingdom (UK) [25, 26], United States (US) [27–29], Denmark [30], Jordan [31], Lebanon [32] and Turkey [33], reflecting patients’ acceptance and interest in alternative approaches to infertility treatment. Herbal supplements and acupuncture, used to supplement or replace ART, are perhaps the most studied infertility approaches [34, 35]. The range of CAM modalities, treatments and emphasis on individualized therapies however, limits assessment of the therapeutic efficacy of CAM to treat infertility [13, 33, 34, 36]. About 65-75% of Australian infertility patients report use of CAM [14, 22], compared to 29% of US patients [27], and 40% of UK patients [25] indicating regional differences in CAM uptake. Use of herbal supplements during pregnancy also exhibits regional variation, with use most common in Russia, Eastern Europe and Australia [37]. In Canada, 9-23% women [37, 38] report use of herbal supplements during pregnancy while 31% of male infertility patients acknowledged use of alternative therapies including vitamins, minerals and herbal remedies [24]. These studies indicate that Canadians are using CAM for reproductive health, however the role of Canadian CAM practitioners in infertility treatment has not been examined.

Despite lack of evidence regarding CAM efficacy, patients are increasingly using CAM to replace or supplement ART. CAM modalities may be useful to help patients mitigate lifestyle risks to improve fertility and ART success. The attitudes and experiences of CAM practitioners regarding their roles in infertility treatment and perspectives on infertility patients’ motivations have not been well studied. To address these gaps, we have examined the roles of CAM professionals, practicing in Ottawa, Canada, in the treatment and support of infertility.

## Methods

### Recruitment

CAM practitioners were initially identified through assessment of Ottawa, Canada CAM practice websites and contacted to participate in this study. This purposive recruitment strategy primarily targeted practitioners with an online (website) or advertised presence in the community, thereby identifying established practices. Recruitment included a brief summary of the research project, mechanisms of participation and an advance copy of the informed consent form, which each practitioner signed on the day of the interview. Participation was assessed on the basis of CAM practice in the Ottawa, Canada region and previous experience treating or supporting infertility. Ten Ottawa-area CAM practitioners represented the study sample. This study was approved by the University of Ottawa Research Ethics Board.

### Data collection

Individual interviews with CAM practitioners, conducted March-November, 2011, were audio-recorded and noted, followed by transcription. One interview, lasting 45-60 minutes, was conducted with each participant at their practice. Interview topics enabled CAM practitioners to describe their perspectives on infertility and their roles in infertility support and treatment (Table 1). Demographic data were also collected (Table 2).

**Table 1:** **Interview questions**

Topic	Interview questions
Models of infertility	How would you describe infertility?
Experiences from CAM practice	Please discuss how [naturopathy/homeopathy/acupuncture…etc.] is used with your infertility patients.
	Is [naturopathy/homeopathy/acupuncture…etc.] used to support infertility or directly treat infertility?
	Do you observe gender difference in terms of your infertility practice?
Practitioners’ perceptions of CAM-infertility patients	What, in your opinion, motivates patients with infertility to seek additional support through complementary and alternative medicine?
	Related to infertility, what would you describe as the greatest unmet need for these patients?
Collaborations	What is your relationship with physicians who treat infertility using medical models?
Recommendations	Recommendations for improvement?
	What recommendations would you make regarding emotional support for infertility?

**Table 2:** **Participant characteristics**

CHARACTERISTIC	NUMBER
Males	3
Females	7
**AGE (years)**	
<30	1
30-39	6
50-60	3
**PROFESSIONAL TITLE**	
Naturopathic Doctor	5
Acupuncturist	2
Doctor of Oriental Medicine	1
Hypnotherapist	1
Medical Doctor	1
**DOMAIN SPECIALITIES**	
Naturopathy	6
Homeopathy	1
Acupuncture	9
TCM/Oriental Medicine	6
Medicine	2
Hypnotherapy	2
**PROFESSIONAL EXPERIENCE**	
0-5 years	4
6-10 years	2
11+ years	4
**LICENSING**	
BDDT-N	4
CPSO	1
American NCCAOM	2
CAATCM	1
Unspecified	2
**% OF PRACTICE DEDICATED TO INFERTILITY**	
≤5%	3
15-25%	4
40-60%	3

*Abbreviations*: *TCM* Traditional Chinese Medicine, *BDDTN* Board of Directors of Drugless Therapy- Naturopathy, *CPSO* College of Physicians and Surgeons of Ontario, *NCCAOM* National Certification Commission for Acupuncture and Oriental Medicine (US), *CAATCM* Canadian Association of Acupuncturist and Traditional Chinese Medicine.

### Data analysis

Interview data were systematically explored using qualitative content analysis; a method which serves to summarize the data content [39]. Ten interview transcripts were coded for content themes using NVIVO™ (QSR International, Cambridge, MA, USA). Briefly, the coding process involved identification of major themes which were labelled and organized using NVIVO™ a qualitative data analysis software program. Themes emerged inductively with preliminary categorization provided by interview topic. Major themes were identified as concepts, ideas or perceptions expressed by at least five of the ten participants. Coding meetings, which included the interviewers, provided opportunities to refine thematic categories, ensure coding consistency and confirm saturation [40, 41]. The coding and analysis process occurred while interviews were ongoing, such that recruitment was terminated when saturation was achieved.

## Results

### Participants

Seven female and three male CAM practitioners participated in this study (Table 2). Most participants engaged in interdisciplinary practice with multiple treatment domains. Acupuncture, naturopathy and TCM were most common.

### Perspectives on infertility

Participants described a biomedical model of infertility which recognized psychosomatic stress and lifestyle determinants. When asked to describe infertility, four major themes emerged: *inability to get pregnant, time-clinical model, stress as cause of infertility, biological model of infertility*.

*“We just use the Western definition of a woman in her mid-thirties or below, trying to conceive for one year and not being successful. We could consider that infertile. Or older women, we generally give them six months of effort and if that does not come about, we would consider them infertile.” CAM201110, male doctor of Oriental medicine/acupuncturist*

*“I believe that the base of infertility, as is the base of most things, comes from psychosomatic roots. The female body knows how to get pregnant.” CAM201105, female hypnotherapist*

*“Our training includes the biological model so we use that, at least as a starting point. Beyond things like physiology and endocrinology, we also assess lifestyle factors such as stress, the environment, exposure to toxins, relationship issues, and previous medical history.” CAM201103; female naturopathic doctor (ND)*

### Integrated approach to infertility

CAM practitioners used interdisciplinary, individualized, holistic infertility treatment approaches with patients supported by stress and lifestyle management. Diagnostic investigations often included physical assessments, use of lifestyle-clinical history questionnaires and fertility hormone laboratory tests. Major themes included: *individualized approach, stress management, lifestyle management, holistic approach, interdisciplinary, biological approach.*

*“I think the integrative approach would definitely include treatment of psycho-emotional stress. It would include a number of natural health products that are aiming to restore hormone balance. So I think like something like acupuncture or meditation practice or even emphasizing regular exercise. You know very very simple things that would improve health and thereby improve fertility secondarily.” CAM201108; male, medical doctor (MD)*

*“Naturopathic medicine, the way that we’re regulated in the province of Ontario, we assess each patient individually. So we do a full biomedical, physical assessment. We check things like blood pressure, heart rate, we do a full screen, physical exam and we can order lab work. So most of my fertility patients, if they aren’t coming with labs from a reproductive endocrinologist already, that’s typically something I will ask them to get done or will requisition from here.” CAM201103; female ND*

*“But what works for one patient will not work with the other, because no two trees are the same, no two systems are the same. If I have a hundred patients with infertility, I end up doing probably ninety-five different treatments… almost everyone needs a unique remedy” CAM201106; male ND/MD/homeopath*

### CAM infertility patients

CAM patients were described as predominantly female and motivated to become pregnant. Participants’ perceptions of gender and infertility practice produced three major themes: *most patients are women, women are more open to CAM, men are resistant to CAM.* Participants perceived that in general, women were more receptive to CAM for infertility and other health issues.

*“I think my practice in general is mostly women. I think it has to do with the fact that women are just more proactive with their health. They are just more in tune with their bodies and they sort of buy into the naturopathic paradigm more because I think they believe that its value in preventing disease and working on deeper issues versus just symptomatic improvement. I think that a lot of men want the quick fix - you know, the pill.” CAM201104; female ND/acupuncturist*

*“I usually see a lot more female than male, because I think men usually don’t take care of themselves- they neglect their health and they don’t like to talk. I think 75% of my practice are female, regardless of what disease they have. And, those men that come it’s because their wife has forced them to come. Also, because alternative medicine is a lot more accepted by women than by men. Men are very engineered mind, very mechanical in their approaches - they have a very hard time to believe that something that conventional medicine has not put their seal of approval is of any value. Women are not like that. I think women are a lot more open to explore and investigate”, CAM201106; male ND/MD/homeopath*

Participants were asked to consider their patients’ motivations for choosing a CAM approach. Major themes included: *women will do anything to get pregnant; end of line reached with conventional medicine; identified CAM via internet or word of mouth*.

*“And usually, with fertility in particular, most women are- will do almost anything at the point that they come in to see the naturopath. You can ask them to fly to the moon and back and they would try if they could.” CAM201101, female ND*

*“So often times I see people, it tends to be around this two year mark where, as you say, there is not a lot of research on to what is an alternative besides my doctor and what those recommendations are. So often times, I see them when they are sort of at the end of their rope thinking like, “oh, my goodness, is there ever going to happen for me”, and there is a lot of emotional strain and stress around it.” CAM201105, female hypnotherapist*

*“Yeah…I think that’s more what people do these days –Internet searches… we ask them where they got our name from and you know most of the time it’s through Internet searches or referral from a fertility clinic.. sometimes it’s word of mouth.” CAM201109, female, acupuncturist, doctor of Chinese medicine*

### Infertility practice collaborations: CAM and Conventional medicine

Participants described limited professional collaborations with physicians (general practice, gynecologists, fertility specialists) who treat infertility. Although two CAM practitioners were actively collaborating with the local fertility clinic, most participants’ interactions with conventional medicine were limited to CAM practitioners’ requests for patient files and laboratory test results. Major themes included: *no medicine collaborations, open to collaborations.*

*“I basically don’t have a relationship with any of them. I just.. communicate with the fertility clinic via faxes. I will send for request for records and they’ll send them over and they are very amenable to that. I have never had issues with getting records, so they are very open, but I have never met any of them, never spoken to any of them. Absolutely no relationship.” CAM201104, female, ND/acupuncturist*

*“I haven’t had any referrals yet, but the longer I am here in Ottawa the more people I will meet… I would like to go down and meet them because the more that they* [fertility doctors] *get to know me and know my skill set the more that there will be referrals there. However to date there aren’t any.” CAM201102, female, ND*

*“If the client wants, you know, we go to the fertility center and immediately before they have their medical procedure done, we do* [acupuncture] *treatment at the center and immediately after the treatment is done, we do a post-treatment at the center.” CAM201110, male doctor of Oriental medicine/acupuncturist*

Improved CAM-medicine collaborations were recognized as beneficial to patients. Four participants suggested an *integrated conventional medicine-CAM approach. Communication, awareness* and *education* were each identified by three participants as mechanisms to improve collaborations.

*“What would be ideal would be more of an integrative approach as a whole so to have your conventional fertility specialist working even in the same building, the same office, as a naturopath or other complementary, you know, or to have a system in place where they could do both” CAM201101, female ND*

*“Well I think that some sort of formal line of communication between fertility doctors and alternative providers is a really good idea. I would be willing to contribute to that if something gets started.” CAM201108, male MD*

*“I’m sure a part of it could come from my end and you know with other family doctors I will often send a letter of introduction just to kind of let them know that I am not there to take business away or I’m just there to support the patient. I think probably education on their side, just in school, in terms of understanding what a naturopathic doctor does. I think a lot of them are skeptical because they just don’t know anything about it.” CAM201104, female ND/acupuncturist*

### Gaps in infertility patient care

CAM practitioners identified *lack of emotional support* as the major unmet need for infertility patients. *Education around lifestyle management, social networks* and use of an *integrated approach* were also mentioned.

*“Emotional support. I would say. Oddly enough, there’s a lot that conventional medicine can do to push hormones and you know, push ovulation, but where I see that part is lacking is really in the emotional support that people need.” CAM201101, female ND*

*“More information on how lifestyle really plays a factor in fertility. How important it is to keep ourselves healthy and to develop a healthy lifestyle so that when we are ready to conceive, the chance it will happen faster. I think more emotional support would definitely be beneficial, particularly with the stress around conceiving because when people get in phases where they are experiencing high levels of stress and anxiety, that’s another confounding factor that can play a road block in terms of healthy conception” CAM201102 female ND*

Although participants perceived that their individualized, holistic approach mitigated some of the stress associated with infertility, they noted that for some patients formal emotional supports *(counseling or support groups)* would be required.

*“If they are having a really hard time with it, definitely see a counselor. Acupuncture can be really good to get rid of like emotional blockages. And talking to their partner. Be really open as well”. CAM201107, female acupuncturist.*

*“I would say have a therapist that they can access, or have that as part of their system. I mean certainly there are psychologists and people that are covered under OHIP* [Ontario Health Insurance Plan] *and so would fall under the medical model. I think, of course, always, the ultimate would be to have everybody working together under the same roof and have people who are particularly specialized in the field. Always an integrative approach.” CAM201101, female ND*

## Discussion

Ten Ottawa CAM practitioners willingly described their holistic, interdisciplinary and individualized approaches to infertility treatment and support. CAM practitioners recognized biological determinants of infertility and their interactions with environment, lifestyle and stress. CAM practitioners readily described physiological anomalies as causes for illness, disease and disability, acknowledging that physiological systems are perturbed through modulating effects of lifestyle and stress. Treatment plans were predicated on fertility centre diagnostic results, blood/urine laboratory findings, physical examinations along with patient lifestyle factors. A biological model of infertility was most strongly proposed by participants with training in naturopathy who also emphasized patient education and lifestyle risk mitigation and prevention. All participants considered that individualized treatments along with lengthy appointments fostered patient disclosures on sensitive issues including poor lifestyle habits, stress and family relationships. Australian CAM practitioners who specialize in women’s health reported similar individualized, holistic approaches to infertility; appreciated by patients as positively reinforcing the provider-patient relationship [18]. The patient-centered, holistic, personalized treatment approach central to acupuncture and other CAM therapies has been credited with development of patients’ trust, sense of personal control and empowerment [18, 42, 43].

Ottawa CAM-infertility patients were characterized by practitioners as predominantly female, extremely motivated to become pregnant and open to alternative treatments. CAM-infertility patients are typically older, female with a relatively high socioeconomic status [14, 44]. Participants perceived that CAM fertility-related investigations were driven by the female partner, with men initially reluctant participants. Although men and women experience infertility differently, men express strong desires to conceive and subsequent grief with infertility [45–47]. Ottawa providers perceived infertility patients’ motivations for CAM treatment to be related to their strong desire to achieve pregnancy and dissatisfaction with ART, consistent with previous studies [18,20,27]. The Internet, word of mouth and previous experiences with CAM were believed to contribute to patients’ awareness of CAM providers’ services.

Ottawa practitioners recognized infertility and ART as significant contributors to patients’ emotional distress. Patients diagnosed with unexplained infertility are at particular risk for depression, distress and difficulty reaching acceptance of their infertility [46, 47]. Discontinuation of ART is not only associated with financial, relationship and psychological stressors [12] but the burden of treatment itself [48]. Physical pain and discomfort, adherence to injection protocols along with clinic environmental factors (e.g. poor organization, depersonalized care, limited time for discussion) may all contribute to infertility patients’ dissatisfaction with ART [48]. Ottawa CAM practitioners identified their individualized approach and lifestyle management as mitigating some of their patients’ fertility-related emotional distress. Integration of formal counseling during the infertility treatment process and for patients who struggled with more debilitating symptoms was also recommended. UK, Australian and New Zealand acupuncturists also recognized the significant emotional toll of infertility treatments on patients, alleviating distress through CAM and patient support [23, 42, 43].

Interactions with conventional medical doctors who treat infertility were generally limited to requests for patient records. Although Ottawa CAM practitioners were receptive to CAM-medicine collaborations, they acknowledged several barriers including lack of awareness and understanding of CAM approaches and perceived negativity to CAM, consistent with previous studies [18, 43]. The paucity of randomized control trial studies to properly assess the efficacy of CAM treatments for infertility [36] contributes to the often negative perception of CAM by conventional medicine [49], however, it is also evident that patients are choosing CAM for infertility support and treatment [18–24]. Indeed, UK patients pursued CAM for fertility enhancement in spite of their skepticism about its efficacy [25]. Similarly, Australian infertility patients opted for CAM despite being unsure of the safety testing standards or regulatory approval for CAM remedies [21]. Infertility patients often do not disclose CAM use to their medical providers because the topic is not introduced, perceived lack of relevance or concerns that physicians would have negative attitudes towards CAM [14, 18, 21, 22, 24, 29]. CAM use may be relevant in ART outcomes, as demonstrated by a prospective study of Danish infertility patients which reported that concurrent CAM use was associated with a 30% decrease in ongoing pregnancy and live birth rates [30]. Although for some infertility patients treatment failure and ART discontinuation may influence the choice to use CAM, it is essential that current patients discuss CAM use with physicians due to the potential for some alternative therapies to interact with ART [29, 30]. This issue was recognized by Ottawa CAM practitioners, particularly those with active ART collaborations. In spite of physicians’ concerns regarding the efficacy of CAM and lack of scientific evidence [13, 19, 29], increasingly conventional medicine recognizes the holistic, patient-centered approaches of CAM to be beneficial [13, 29].

In North America, CAM is primarily used to supplement conventional medical care, rather than as an alternative [16, 17, 50], suggesting that patients desire evidence-based health care that is also holistic, patient-centered and individualized. Ottawa CAM practitioners asserted the patient-benefits of a more integrated CAM-conventional medicine approach to fertility treatment; also expressed by Australian infertility patients [20]. Physicians’ development of CAM practice skills such as patient rapport, attentiveness, listening and counseling, would greatly improve perceptions of patient care [13, 25]. Many aspects of women’s reproductive health, including menopause, infertility and pregnancy, have been identified as ideally treated by an integrated CAM-conventional medicine approach [51]. Credentialing CAM providers, faculty development, education and cultural sensitivity regarding the philosophies of CAM models are general strategies to enhance CAM-conventional medicine collaborations [52]. Encouragingly, integrated models have been developed in nine North American academic medical centers which combine research, CAM-conventional medicine clinical care and education [53].

### Limitations

The limitations of our study include small sample size and the potential participant bias of self-selection. Our participant sample was limited to practitioners with established clinic practices as identified through websites or by referral. Participant characteristics including infertility patient experience, years of CAM practice experience, CAM treatment domains, practitioner training, age and gender yielded a heterogeneous sample, however responses to interview topics were fairly consistent and reached saturation.

## Conclusion

This qualitative study enabled an in-depth exploration of CAM practitioners’ support and treatment of infertility. CAM treatment models recognized biological, environmental and psychosomatic impacts on infertility. Patient relationships were established using a holistic, individualized approach, which may mitigate some of the emotional distress associated with infertility. It is anticipated that a greater understanding of CAM approaches to infertility support and treatment will enhance cross-professional relationships for integrated infertility patient care.
